# Methodological factors affecting joint moments estimation in clinical gait analysis: a systematic review

**DOI:** 10.1186/s12938-017-0396-x

**Published:** 2017-08-18

**Authors:** Valentina Camomilla, Andrea Cereatti, Andrea Giovanni Cutti, Silvia Fantozzi, Rita Stagni, Giuseppe Vannozzi

**Affiliations:** 1 Department of Movement, Human and Health Sciences, University of Rome “Foro Italico”, Piazza de Bosis 15, 00135 Rome, Italy; 2 Interuniversity Centre of Bioengineering of the Human Neuromusculoskeletal System, University of Rome “Foro Italico”, Piazza de Bosis 15, 00135 Rome, Italy; 3 Information Engineering Unit, POLCOMING Department, University of Sassari, Viale Mancini, 5, 007100 Sassari, Italy; 4 Department of Electronics and Telecommunications, Politecnico di Torino, Corso Castelfidardo, 39, 10129 Turin, Italy; 5 Centro Protesi INAIL, Production Directorate - Applied Research, Via Rabuina 14, 40054 Vigorso di Budrio (BO), Italy; 6 Department of Electrical, Electronic and Information Engineering “Guglielmo Marconi”, Alma Mater Studiorum University of Bologna, Via Risorgimento 2, 40136 Bologna, Italy

**Keywords:** Inverse dynamics approach, Kinematics, Dynamics, Joint moments, Stereophotogrammetry, Gait analysis methodology, Reproducibility, Reliability, Sensitivity, Error propagation

## Abstract

Quantitative gait analysis can provide a description of joint kinematics and dynamics, and it is recognized as a clinically useful tool for functional assessment, diagnosis and intervention planning. Clinically interpretable parameters are estimated from quantitative measures (i.e. ground reaction forces, skin marker trajectories, etc.) through biomechanical modelling. In particular, the estimation of joint moments during motion is grounded on several modelling assumptions: (1) body segmental and joint kinematics is derived from the trajectories of markers and by modelling the human body as a kinematic chain; (2) joint resultant (net) loads are, usually, derived from force plate measurements through a model of segmental dynamics. Therefore, both measurement errors and modelling assumptions can affect the results, to an extent that also depends on the characteristics of the motor task analysed (i.e. gait speed). Errors affecting the trajectories of joint centres, the orientation of joint functional axes, the joint angular velocities, the accuracy of inertial parameters and force measurements (concurring to the definition of the dynamic model), can weigh differently in the estimation of clinically interpretable joint moments. Numerous studies addressed all these methodological aspects separately, but a critical analysis of how these aspects may affect the clinical interpretation of joint dynamics is still missing. This article aims at filling this gap through a systematic review of the literature, conducted on Web of Science, Scopus and PubMed. The final objective is hence to provide clear take-home messages to guide laboratories in the estimation of joint moments for the clinical practice.

**Electronic supplementary material:**

The online version of this article (doi:10.1186/s12938-017-0396-x) contains supplementary material, which is available to authorized users.

## Background

**Fig. 1:**
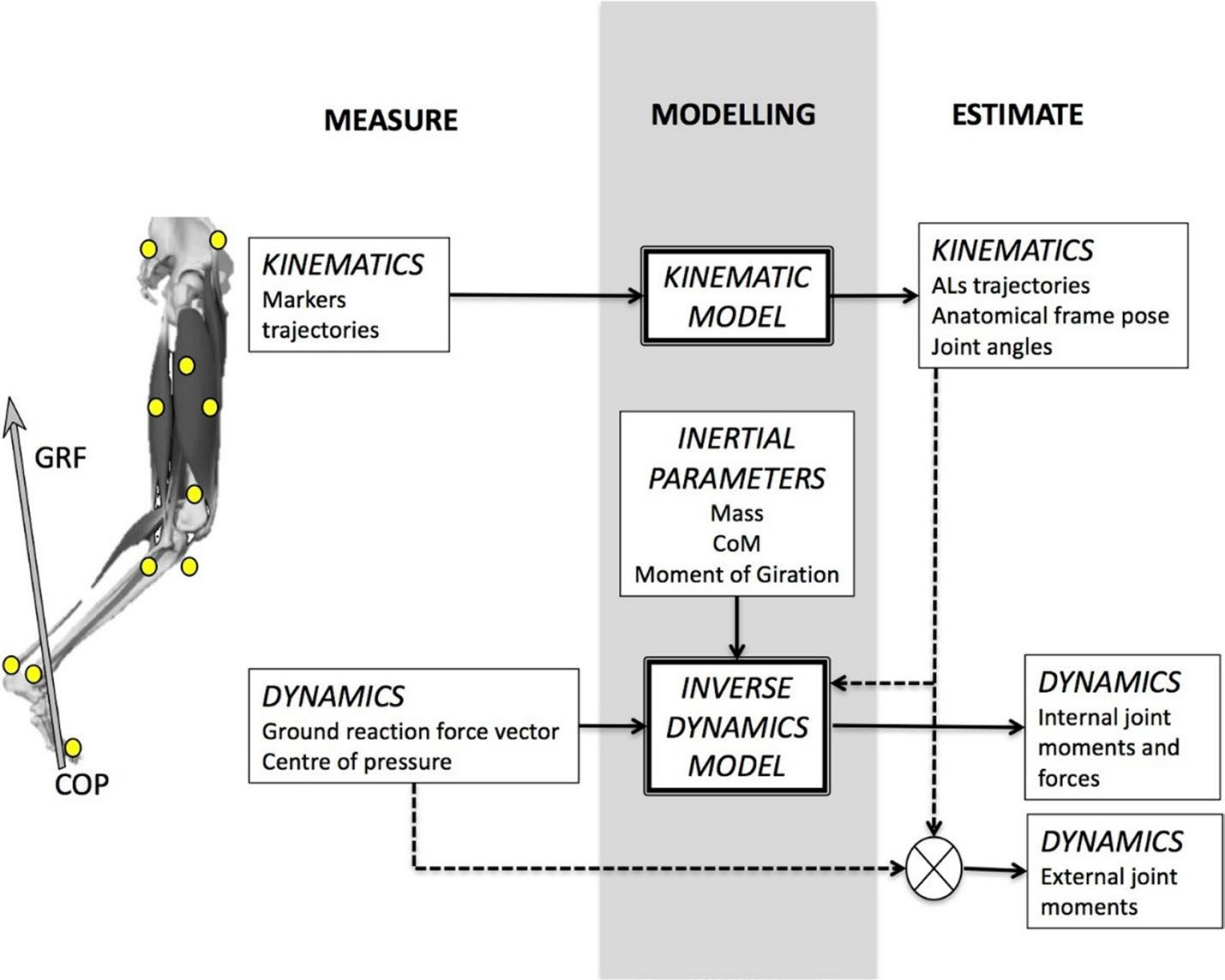
Schematic depiction of the workflow to estimate joint dynamics from measurements of kinematics and ground reaction forces (GRF), through kinematic and dynamic modelling of the body, and inertial parameters (inverse dynamics approach—IDA). The alternative estimation of external joint moments from kinematics and GRF is also reported

Quantitative motion analysis provides an objective description of joint kinematics and dynamics. It is recognised as a useful tool in clinics for functional assessment, diagnosis, planning of therapeutic and rehabilitative interventions, and outcome evaluation. In these applications, ensuring an accurate and reliable estimation of 3D joint moments is crucial. The most relevant sources of error affecting the estimation can be identified by reviewing how 3D joint dynamics is calculated. Two alternative methods are commonly applied (Fig. 1).

The first is the so-called inverse dynamics approach (IDA): the human body is represented as a multi-body chain of rigid segments, and Newton–Euler mechanics is applied iteratively to each segment to calculate net internal joint moments and forces [1–4]. *Marker trajectories* from stereophotogrammetry and *ground reaction forces* (GRF) from dynamometric platforms (“force platform” in short), are the input to the *kinematic* and *dynamic models* of the human body.

In general, the resultant joint moments acting at a joint are generated by a combination of muscle, ligament, and joint contact forces [5]. However, in almost all clinical gait analysis protocols, human joints are represented by either ideal spherical or hinge joint whose centre/axis approximates the joint centre/axis of rotation. Within this modelling assumption and in the hypothesis that friction is negligible, the resultant of the bone-to-bone contact forces passes very close to the geometrical centre/axis of the joint and therefore its contribution to the net moment is commonly neglected. Therefore, as first approximation, it can be assumed that the resultant moment of the intersegmental forces represents an estimate of the overall muscular moment plus the contribution of the ligaments. Following this description, the specificity of bi-articular muscles is disregarded [6].

IDA also requires the estimation of the *body segment inertial parameters* (BSIP) (i.e. mass, position of the centre of mass in the segment coordinate system, moments of inertia), usually obtained for a specific subject from generalised anthropometric tables.

A *second* simplified method requires only segmental/joint kinematics and GRF to estimate external moments. This approach neglects the contribution of the segment inertial and gravitational forces, assuming all the mass concentrated in the body centre of mass, and provides an estimate of joint moments only during the stance phase of gait (when GRF is measured) with minimal computational effort. Despite of its extreme simplicity, this approach has been applied in clinical gait analysis studies, and provided reasonable estimations of joint loadings during the stance phase, when the inertial contribution is minor [7], particularly for distal joints (ankle and knee) [1].

It follows that the sources of error can be summarized in four groups: (1) kinematic measures and processing, (2) measure of the GRF and processing, (3) determination of joint model parameters, and (4) estimation of the inertial parameters.

Kinematic errors include, for instance, the errors intrinsic in the measurement system, the soft tissue artefact and the inaccurate localization of anatomical landmarks, with the latter two recognized the most critical [8, 9].

GRF is measured by force platforms as the resultant mechanical interaction between the foot and the ground, described in the form of a 3D force vector applied in the centre of pressure (COP), represented in the platform coordinate system. Force platforms are prone to measurement errors per-se, but their calibration in the stereophotogrammetric coordinate system should also be regarded as a potential source of inaccuracy.

Errors comprised in joint parameters include the position of the joint centres, and the position and direction of the joint axes of rotations. The latter errors have an effect on both joint kinematics and dynamics [10, 11].

Finally, BSIPs can be estimated using several methods, namely predictive equations based on measurements of cadavers or living subjects [12–17], geometric approaches [18, 19], data of living subjects obtained through medical imaging technologies [20–23], or estimates provided by the solution of a non-linear optimization problem [24–26].

Numerous studies analysed, separately or in conjunction, the influence of these sources of error on the estimation of joint moments, but a systematic review of their impact on the clinical interpretability of results is missing. The present systematic review aims at filling this gap by investigating which of the aforementioned factors influence the estimation of joint moments to a greater extent. Whenever possible, implications on sensitivity, reliability and reproducibility of data for clinical use were also addressed.

## Methods

### Articles selection

#### Inclusion and exclusion criteria

We included studies published in English as full papers, using stereophotogrammetry and force platforms as measurement systems, analysing gait and activities of daily living (Table 1). Subsequently, we excluded articles not analysing the impact on joint dynamics of kinematics measurements and processing, GRF measurements and processing, joint kinematic and dynamic modelling, and body segment inertial parameters.

**Table 1:** Inclusion criteria considered for the current systematic review

Criteria	Definition
Measurement instruments	Stereophotogrammetry and force platforms
Body area	Lower limbs
Motor tasks	Gait and selected activities of daily living (stair, chair, squat)
Areas of interest	Kinematics measurements and processing Force measurements and processing Joint model parameters Body segment inertial parameters
Publication type	Journal papers in English
Cohort under investigation	Healthy and able-bodied human subjects

#### Search strategy

Articles were searched in Web of Science, PubMed, and Scopus (until February 9, 2017). Keyword search was performed to match words in the title, abstract, or keywords fields. A first general search was performed selecting keywords to define subjects, general topics of interest and motor tasks. Four subsequent refinement searches were performed for kinematics (V.C. and G.V.) and force (R.S.) measurements and processing, joint parameters (A.Ce.), and body segment inertial parameters (S.F.). All Boolean researches are reported in Appendix.

#### Review process

Each reviewer first removed conference proceedings, theses, and duplicate journal references. Secondly, title and abstracts of the remaining papers were evaluated for inclusion based on the relevance to the four areas of search. A full text evaluation was performed if the title and abstract failed to provide adequate information. Finally, a manual screening of the reference lists of all included studies was undertaken to include further eligible studies not retrieved during the systematic database search.

#### Reliability, reproducibility and sensitivity analyses

In the following sections, the terms ‘‘agreement’’, ‘‘reliability’’, ‘‘reproducibility’’, and ‘‘repeatability’’ will be routinely used. As previously highlighted by Bartlett and Frost [27], these terms have been frequently abused in the literature. We therefore decided to strictly adhere to the definitions reported in [27], which are summarized here for convenience:

- *Repeatability* refers to the variation in repeated measurements made on the same subject under identical conditions, e.g., same method and same rater;
- *Reproducibility* refers to variation in measurements made on a subject under changing conditions, e.g., using different methods. When the changing condition refers to two different methods, this specific type of reproducibility study is commonly referred to as ‘‘method comparison study’’;
- *Reliability* relates the ‘‘magnitude of the measurement error in observed measurements to the inherent variability in the underlying level of the quantity between subjects’’ [27]. Therefore, reliability depends upon the heterogeneity of the population in which the measurements are made. The typical parameters used in reliability analysis are the intra-class correlation coefficients (ICC) or the coefficient of multiple correlation (CMC) widely adopted in human movement analysis [28];
- *Agreement* ‘‘quantifies how close two measurements made on the same subject are, and is measured on the same scale as the measurements themselves.’’ Agreement is an intrinsic characteristic of the method(s) and does not depend on the population in which measurements are made, unless bias or measurement precision varies with the true value being measured. Therefore, the measured agreement does not typically need to be recomputed when considering pathological groups, if the distribution of measurement errors is uniform across the range of true values.

To compare the results provided by the different studies, joint moment values were also expressed in % BW * H thus obtaining dimensionless quantities whenever possible. When no information about the subject/s height was reported in the original articles, a reference height of 1.7 m was considered. The results obtained after conversion were reported within brackets and were rounded to the nearest decimal place.

## Results

### Review selection and identification

**Fig. 2:**
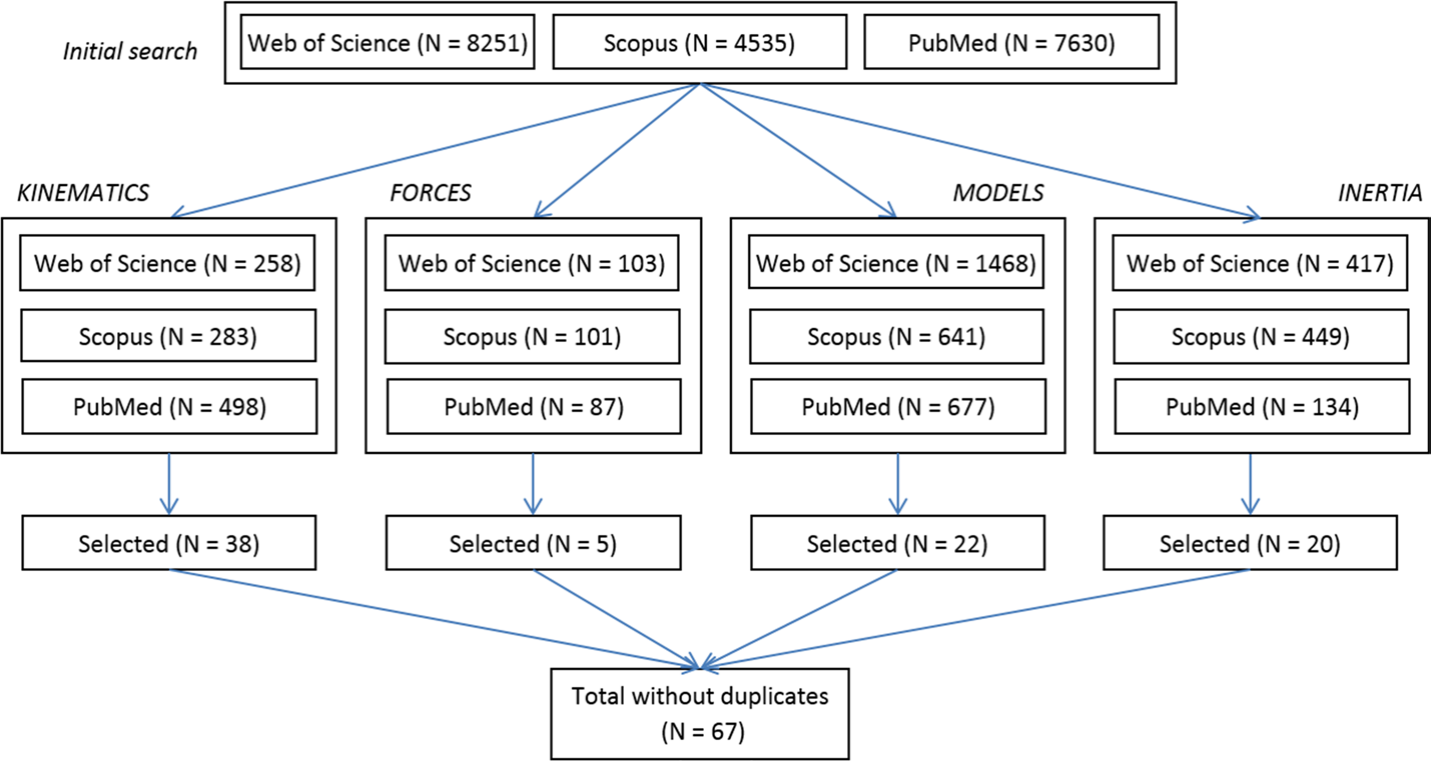
Search strategy flow chart. After running the general search, the three researched databases listed 8251, 4535, 7630 papers, respectively (including duplicates). Subsequent refinement yielded to a total of 1039 results for kinematics and processing, 291 for GRF measurements and processing, 2786 for joint models, and 1000 for BSIP, respectively. After examining the title, abstract or full text, only 38, 5, 22, and 20 remained. The overall total, without counting articles included in more than one area of analysis and duplicates, was 67 papers

The initial search yielded 8251 (Web of Science), 4535 (Scopus) and 7630 (PubMed) results (Fig. 2). Over the three search engines, subsequent refinement yielded to a total of 1039 results for kinematics and processing, 291 for GRF measurements and processing, 2786 for joint models, and 1000 for BSIP, respectively. Selection performed separately for each area and based on title and abstract or full text, lead to 38, 5, 22, and 20 journal papers. Despite considering studies conducted on healthy subjects only, the search reported 6 studies including patients [5, 29–33] whose conclusions were valid independently from the population of interest and were retained for further analysis. After excluding duplicates, 67 papers were finally listed.

Of the 67 papers, 11 were included in more than one area and were analysed multiple times according to the focus of the analysis, but were included only once in Additional file 1: Appendix S1 (on-line material), where the studies identified for inclusion in the systematic review are listed.

### Kinematics

The reviewed articles suggest that interactions exist between joint kinematics and the resultant joint moments, the measured/estimated kinematics being indeed one of the principal causes of uncertainty in dynamics estimations [34]. Kinematic errors typically include: *apparent* marker movements (due to errors in marker reconstruction performed by the optical systems), and *real* marker movements (due to the interposition of soft tissues between markers and the underlying bone, the so-named soft tissue artifact—STA) [9]. Another source of uncertainty is represented by the *identification of anatomical landmarks* which, being a subjective procedure, is prone to repeatability issues [8]. Overall, this source of error introduces uncertainties that can assume a considerable importance (6–232% of the estimated torque magnitude: 0.005–0.03% BW * H), these magnitudes being smaller for more distal joints [35].

In this section, we analyze the influence on IDA results of these three factors, as well as the role of the *bone pose estimation and the definition of the coordinate systems*, and of the *gait protocol adopted*.

#### Measure of marker trajectories

The literature remarks that noise affecting marker trajectories represents the first source of error to be compensated, due to its amplification/propagation during differentiation to calculate velocities and accelerations. This issue is particularly important when dynamics is estimated using kinematics only, without GRF measures [36]. Several methods were described for its compensation: digital filters, splines, spectrum analysis techniques [37], Kalman smoothing and multi-body kinematics optimization [38]. A sensitivity analysis was performed in [39] and it was found that filtering kinematic data with a cut-off value of 4.5 Hz improves gait dynamic estimates.

#### Soft-tissue artifact

Few studies quantified the effect of soft tissue artifacts of thigh and shank on the knee moments during walking [40, 41], stair ascent [42] and sit-to-stand [43] against gold standard measurements. Knee extensor and abduction moments were significantly underestimated in the middle of the stance phase of sit to stand, in correspondence to load increase [43]. This result was confirmed during the load transfer phase of stair ascent, but only for knee extension [42]. Similar trends in magnitude were measured for both moments during the single support phase of walking [40, 41]. Also, Buczek et al. [41] suggested a similar role for the artifact in underestimating the knee extension moment in the same phase, but only inferring it from the comparison of two marker protocols, including and not including a marker on the lateral epicondyle. It was concluded that the magnitude of the observed differences would not likely affect the clinical interpretation of the data [41]. It must be kept in mind, however, that the soft tissue artifact does not only affect pose estimation, but also the determination of the hip joint centre, when estimated through a functional approach [44, 45]. Errors in this determination, as mentioned in the Joint Model Parameter section, may well influence the clinical interpretation.

#### Identification of palpable anatomical features

Three studies focused on errors of palpable anatomical landmark positions and, consequently, of anatomical coordinate systems [38, 46, 47]. Silva and Ambrósio [46] reported that the sensitivity of IDA results to a perturbation in the digitized knee coordinates was associated to errors in the net knee moments of 200-1500 Nm/m on the three axes (corresponding to 0.2–1.2% BW * H). De Groote et al., proved that the uncertainty in locating anatomical landmarks affects joint moments to a larger extent than the uncertainty in BSIPs [38]. Finally, Langenderfer et al. applied a probabilistic method to quantify the effect of the uncertainty in locating anatomical landmarks and BSIPs on joint moments [47]. They concluded that awareness of this uncertainty is crucial in the evaluation of healthy normal and pathologic gait and can improve clinical diagnoses.

#### Bone-pose estimation and coordinate systems definitions

The articles reviewed highlight that the selection of the coordinate systems (CS), despite arbitrary and not an error per-se, influences IDA results and, possibly, their interpretation. This influence can arise both from the definition of the CS used to describe body segment anatomy and the choice of the CS in which joint moments are expressed. If a multi-body (or single-body) kinematics optimization is used, the segment accelerations are also modified. Moniz-Pereira et al. [48] proved that lower limb joint dynamics is sensitive to different pose estimation algorithms, although to a lesser extent than joint kinematics, especially for the frontal and transverse planes (max. RMS difference between algorithms: 0.12 Nm/kg corresponding to 0.07% BW * H (35.4%) vs 11.7° (64%), respectively).

The definition of the shank anatomical CS was proved to influence knee extension and abduction moments: knee extension moments had greater peaks when expressed about an anatomical axis following the line of the malleoli than when the moment was reported about the same axis rotated around the shank longitudinal axis to become parallel to the frontal plane of the subject; conversely, this last choice entails significantly greater first peak abduction moment [41]. Similarly, the choice of different anatomical landmarks (ALs) of the distal femur to define the femoral anatomical CS had an impact up to 25% on the knee flexion–extension moment [49].

For clinical gait analysis, the simple analysis of the sagittal plane moments (2D approach) was often considered appropriate, showing little differences in the overall joint moment patterns when compared to 3D models [50, 51]. Nevertheless, the sagittal view provides only part of the information, especially at the hip level, where abductor moment plays an important role in maintaining trunk balance in the frontal plane.

When a 3D analysis is performed (although all possible CSs for the expression of the net moment vector are mathematically valid), differences in joint moment profiles can be expected depending on the components of this vector being expressed in the global CS or in the proximal, distal or joint CSs [50, 52–57]. It can be argued that joint moments expressed in local CSs may allow interpreting the trajectories in terms of the moments that cause them [58]. Indeed, for able-bodied adult gait, joint moments represented in the global CS and in different anatomical CSs (being it either proximal or distal or a non-orthogonal joint CS), presented significant differences primarily for the transverse and frontal plane joint moments (resulting in about 5% BW * H variability due to CS for key peak moments of hip, knee, and ankle) [56, 57]. Further differences in the transverse and frontal plane joint moments may arise for simulated [55] or actual pathological gait (resulting in about 20% BW * H variability due to CS for key peak moments of hip, knee, and ankle) [56]. Similar results were obtained in [50]: based on a larger adult sample, differences arose also in the frontal plane, with global CSs generally underestimating adductor muscle moment. Frontal plane moments are also influenced at joint CS level by whether the adduction moment axis follows or does not follow the internal/external rotation of the lower limb [33]. At the ankle level, only for frontal plane the global CS proved to overestimate the invertor moment at midstance and underestimate the late stance evertor moment [54]. Using non-orthogonal CS, either based on the axes used to describe joint kinematics [52, 53], or on generalized coordinates [46, 59], may yield better anatomical insight on the joint structures involved with the joint dynamics. In both cases, the final results depend on whether an orthogonal and non-orthogonal projection of the joint moment on the axes of joint CS are used [13, 52, 53, 60].

Care should, therefore, be taken whenever comparisons between studies are made in which the anatomical CS axes used to define the bone pose or to represent the joint moments are not the same. These differences may influence the clinical interpretation according to the parameter under analysis [33], although statistical techniques, such as principal component analysis, may help in highlighting pathological features that are independent of the coordinate system selection [32].

#### Gait protocols

Several stereophotogrammetric protocols have been proposed for clinical gait analysis [61]; the effect of their differences on joint moment estimation was analyzed during level walking [62–64]. Specifically, excellent intra-session repeatability was obtained for the analyzed protocols, with an excellent reliability in the sagittal plane (CMC > 0.95) and a good reliability in the other two anatomical planes (CMC > 0.67). Kadaba et al. [64] also found a lower repeatability of knee moments with respect to hip and ankle moments. Similar considerations were also extended to stair climbing using the Kadaba’s protocol [65], with joint dynamics more reproducible than kinematics, especially for abduction–adduction and internal–external rotation at all joints. A comparison between the Kadaba’s protocol [64] and a six degrees of freedom model showed that most differences were subtle and unlikely to affect clinical interpretations for normal children, but few substantial differences may deserve further investigation, especially for pathological movements or morphology that may exacerbate model differences [66].

Inter-laboratory consistency of gait analysis measurements using the same protocol is also an investigated issue. Comparison of normative data, as collected using the same protocol in two clinical gait analysis services, highlighted only slight differences in hip and knee extensor moments and all powers, with RMS differences for the inter-laboratory means of less than 0.1 Nm/kg for joint moments (corresponding to 0.006% BW * H), and 0.21 W/kg for powers [67]. Inter-laboratory consistency was also assessed by testing one subject with five different protocols: higher differences were found for kinematics than for kinetics, the latter circumstance being noticeable due to inter-protocol differences, such as the use of standard IDA instead of using GRF for joint moments calculation [35, 68]. In general, differences of 0.5 Nm/kg were pointed out (corresponding to 0.03% BW * H), which are lower than the established minimum detectable change for gait kinematics and dynamics for healthy adults [68].

Changes in the protocols have been proposed to improve IDA calculations, either addressing anatomical calibration or soft tissue artifact. For instance, modified versions of the Davis protocol were proposed using additional markers [69, 70], improving the between-day repeatability [70] and reducing the errors in projecting the joint moment components in the sagittal and frontal planes [69]. Similarly, Petit and colleagues [71] added three proximal shank markers, improving the definition of the proximal shank which, in turn, reduced the knee moment lever arm and lowered the sagittal knee moment. The same reduction was not observed for the knee ab-adduction moment.

Further modifications to gait protocols were adopted for the foot, increasing the number of segments used for its modeling, with the effect of reducing overestimation of ankle joint powers, typical of single-segment models [72], or producing a better description of ankle kinematics in the frontal plane during stance. Improvements in kinematics significantly influenced joint dynamics at the upper levels, in particular the peak hip adductor moment [73]. In both [73] and [72], additional tracking markers for both forefoot and rearfoot were added to the typical cluster tracking calcaneal–tibial motion.

#### Optimized IDA estimates and their implications in clinical gait analysis

Discrepancies in joint dynamics due to different IDA computational approaches has been studied in [74] and knee moment profiles across methods were shown to be different, even though with comparable magnitudes. To overcome this issue, procedures to increase IDA quality were proposed, such as static optimization using a least-square approach, which provided a reduction of about 30% on joint torque errors with respect to the conventional Newton–Euler method [75]. Dynamic optimization models are also used to reconstruct the pose of the body segments under analysis, reducing the consequences of soft tissue artifacts (multi-body kinematics optimization) [38, 76]. This approach, adopting body segment chains with kinematic constraints to model the joints, may be considered adequate for the description of physiological gait. Its adoption is questionable for pathological gait because it alters the joint behavior, that does not follow anymore the kinematic model assumed in the multi-body kinematics optimization.

### Ground reaction force

The number of published papers analysing the propagation of uncertainties in the measurement of GRF is small. Only four papers were found investigating this specific problem [34, 35, 77, 78], of which only two specifically addressed the effect of errors superimposed to GRF measurements [77, 78]. None of them took into account the potential additional measurement errors introduced by the calibration of the force platform in the stereophotogrammetric coordinate system.

When dealing with the estimation of joint dynamics, the majority of the literature considers GRF measurements virtually error-free. Little attention is paid to errors that intrinsically characterize any measurement procedure, and even less attention to the potential effect on the biomechanical variables analysed and the resulting clinical interpretation.

Just like any measurement device, force platforms are characterised by a certain measurement accuracy, declared by the producer in the device data-sheet. According to the data-sheet of common commercial force plates, the expected measurement errors can be bounded between 0.2 and 2% of their Full-Scale Output (FSO). Typical values of FSO in gait analysis are 500 N for force components in the platform plane and 2500 N for the orthogonal component. Although not constant throughout the acquisition, errors in the order of 1–10 N and 5–50 N can reasonably occur in the horizontal and vertical components, respectively, accompanied with errors up to 0.01 m in COP coordinates [34, 35, 77, 78].

When GRF is measured in a gait analysis session, other sources of errors can superimpose to those characteristics of a properly functioning device. Platform calibration errors or inaccuracies, inappropriate setting of the platform (e.g. low threshold, sampling frequency), modifications in the behaviour of the electronic components (e.g. cable interference, contacts, electrical inductance resulting from chances in temperature, humidity or simply aging of the device components) can significantly affect the performance of any force platform integrated in a gait analysis laboratory [79–85]: during in situ testing errors superimposed to COP coordinates were found double than the reference ones [82–84]; accuracy decreases as the point of application of the force moves to the platform peripheries [82], although distributed loads seem to be less affected by this phenomenon than concentrated ones [81]; the minimum vertical force threshold might be up to 113 N in order to estimate the COP within a distance with the declared SD of 0.003 m [84]. Moreover, the performance of the force platform can differ in dynamic compared to static conditions [79]. Due to the relevance of these measurement uncertainties, a number of methods for the in situ assessment of the performance of force platform have been proposed in the literature [79, 84, 85], as well as possible compensation methods [82, 86].

Therefore, the lack of attention paid to the likelihood of errors in force measurement is somewhat surprising [80], particularly in the field of gait analysis, where force measurement data are often proposed as a reference for the gait laboratory quality check [87, 88].

It could be argued that the impact of these measurement errors can be negligible when compared to other sources of error and in terms of their propagation to joint moments. This does not seem to be the case, despite the limited amount of available literature [89]. McCaw and DeVita [77] analysed the effect of errors up to 0.01 m superimposed to COP coordinates in a sagittal model of gait, observing average changes 14% in maximum angular torques (approximately 0.8% BW * H), and up to 13% in the estimation of the flexion–extension transitions time. In their comprehensive analysis of the uncertainties in inverse dynamics solutions, Riemer et al. [35], pointed out that the values of the maximum estimated uncertainties relative to peak joint torque for the ankle, knee and hip are 6–12% (approximately), 50–105% and 114–232%, respectively, depending on the set of perturbations; these uncertainties result from errors superimposed on kinematics, body inertial parameters and force plate measurements, but for the lower body model, the uncertainties in the distance from the COP to the ankle centre of rotation is one of the major contributors. Pàmies-Vilà et al. [34] implemented a similar comprehensive analysis on a 2D model of gait, taking into account uncertainties in the measured force components compatible with those declared in the device data-sheet; the error in the ground reaction torque highly affects the results, up to a normalised root mean square error of 52% in the hip torque; this analysis shows that GRF errors produce higher root mean square errors and normalised root mean square errors than those introduced by inaccuracies in BSIP, but similar to those produced by inadequate kinematic processing. Finally, Camargo et al. [78] analysed the influence of uncertainties in the COP localization on gait dynamics at different velocities, showing that resulting uncertainties on joint moments increase with increasing velocities.

### Joint model parameters

Commonly, human joints are modelled either as spherical or hinge joints. Whereas for the hip joint, the functional consistency between the actual joint and the spherical joint model is almost perfect [90] and therefore a unique centre of rotation exists, this is not true for other human joints. For instance, it was demonstrated that in the knee joint during normal gait the tibiofemoral contact loads contribute substantially to both net extension and adduction moments [5].

Once a convenient joint model is chosen, this has to be tailored for the specific subject under analysis (joint model calibration). Joint calibration procedure is crucial since errors in the parameter determination (joint centre position and axis position and direction) heavily affect the estimated muscular moment arms and consequently the joint moments and their interpretation. Joint parameters are commonly defined by using regressive equations from palpated external anatomical landmarks [64, 91], functional approaches [92–94], multi-body kinematics optimization techniques [95] or bio-imaging techniques [96].

#### Studies classification

The literature introduced the potential benefits of using functional joint centres and axes instead of palpable anatomical landmarks or regressive joint centres to estimate joint dynamics, producing slightly more repeatable hip and knee joint moments [94]. Several studies have dealt with the effects of errors in the joint parameters identification on the estimation of the lower limb joint moments. Since joint moments cannot be directly measured unless implanting instrumented prostheses [5] or using force/moment sensors in prosthetic limb of amputees [97–99], a ground truth is rarely available for evaluation. Therefore, the most common solution is to assess changes in the joint moments patterns due to any changes in possible input data and parameter values. This was accomplished either by directly or indirectly perturbing the joint parameters through mathematical simulations or experimentally by determining the joint parameters using different methods.

In Table 2, a concise classification and description of the relevant literature is provided.

**Table 2:** Details of studies analyzed for the joint model parameters effect on the dynamics estimates

Study typology	Type of analysis	Study	Description
Hip joint			
Mathematical simulation	Sensitivity analysis	[11]	The effects of HJC perturbation in the range ±30 mm on the resultant moments at the knee and hip was investigated via a mathematical simulation during level walking
		[100]	The model parameters of the lower limb were perturbed via a Monte Carlo simulation (joint centres ±10 mm; joint axes ±10°) to evaluate the influence on joint torques
Experimental	Method comparison with gold standard	[29]	Both studies compared the joint moment obtained using generic scaled musculoskeletal model [16] with the HJC location determined using a regressive method [3] and with subject-specific HJC location derived from CT scan
		[30]	
		[103]	This study compared hip joint moments as obtained using standardized X-rays and four non-invasive regressive methods in ten healthy adults during gait
	Method comparison without gold standard	[104]	This study investigated the clinical agreement of four commonly used regression equation sets on 18 healthy pediatric subjects during gait
		[102]	The study tested the influence of different HJC locations, determined according to three regressive methods and a functional method, on hip and knee joint kinetics during a squat exercise on 15 healthy subjects
Knee joint			
Experimental	Sensitivity analysis	[105]	The effects of KJC perturbation in the range ±10 mm in the anteroposterior direction was investigated via a mathematical simulation at different walking speed on 18 healthy subjects
	Method comparison with gold standard	[29]	The knee was described by the planar model and its rotation axis was determined both from skin markers using generic-scaled models and from CT-images of the knee condyles. Eight subjects were analyzed
Mathematical simulation		[100]	The effects of different knee axis inclination on knee joint moment estimation were then investigated. The knee was modelled as a hinge joint and both knee joint centre (±10 mm) and axis (±10°) were perturbed via a MonteCarlo simulation
		[46]	A biomechanical model based on natural coordinates was developed by modelling the knee joint as a revolute joint. Authors conducted a sensitivity analysis by directly perturbing the coordinates of the “knee anatomical point”
Ankle joint			
Mathematical simulation	Sensitivity analysis	[100]	The ankle joint was model as a universal joint (two non-intersecting axes) and the effect of errors on the joint parameters estimation were investigated via Monte Carlo simulation
Experimental	Method comparison with gold standard	[29]	Ankle joint moment obtained using generic scaled musculoskeletal model [16] and with subject-specific ankle joint centre location derived from CT scan. No information about ankle joint and axes determination were provided
Multi-joint			
Mathematical simulation	Perturbation of markers/ALs positions	[47]	A 3D inverse dynamic model of the lower limbs was used to investigate the effect of intra-rater variability in ALs identification. The amount of variability in the ALs identification was described as normal distribution according to experimental observations available in the literature [25]
		[106]	Both studies performed a sensitivity analysis using a highly subject-specific muscolo-skeletal model defined from computer tomography [27] and magnetic resonance [28]. Thanks to the use of high resolution bio-imaging, the uncertainties in the ALs identifications were very small (<2 mm) and therefore errors in joint centres and axes parameters were of one order of magnitude smaller than those observed by using skin markers for ALs locations
		[107]	
Experimental	Inter-session repeatability	[94]	This study compared the repeatability of joint moments obtained from two different gait models (inter- and intra-examiner) on ten healthy subjects. The two gait models differed for the identification of the hip joint centre and the knee flexion–extension axis which in the first case were identified from the skin markers position whereas in the second case using a functional approach
		[51]	The agreement between the joint moments calculated by a 2D and a 3D inverse dynamics models was assessed on 15 healthy subjects during gait recordings. However, since the two models did not share the same joint centres locations differences were expected
		[69]	The study compared the inter-sessions variability of joint moments obtained with the Plug-in-Gait protocol [5] with those obtained using a modified version of it including three extra markers on the medial sides to facilitate the identification of the knee and ankle centres and the calculation of the geometrical prediction of the HJC. Twenty-five subjects were analyzed including 14 healthy and 11 with pathological knee varus alignment
	Method comparison without gold standard	[126]	The study implemented four different body estimators methods: Davis model [127], a kinematic constrained method [76], a single-body optimal method and a multi-body kinematics optimization method [128]. Experimental data were collected on a single healthy adult subject. The effects of kinematic constraints on the estimation of both joint kinematics and moments were analysed in terms of marker trajectories residual, joint dislocation and dynamic residuals. No information on the joint centres or axes as determined by each methods are provided

Results and findings of the relevant studies are critically reviewed following the scheme proposed in Table 2 according to their relevance to the specific joint.

#### Hip joint

The simulation carried out by Stagni and colleagues indicated that errors in the hip joint centre (HJC) location greatly affect hip joint moment [11]. They found that a 30 mm HJC anterior and lateral mislocation caused a mean error of about −22 and −15% in the flexion–extension and abduction–adduction moment components of the corresponding range, respectively (the corresponding values in units of per cent bodyweight (BW) times height (H) are −1.43 and −1.38% BW * H). These errors also produced a delay of about 25% of the stride duration in the flexion–extension moment timing [11]. Reinbolt and colleagues [100] found, based on the Monte Carlo analyses, errors on the hip flexion–extension and hip abduction–adduction moments consistent with those reported by Stagni and co-workers [11]. Similar conclusions, (i.e. overestimation of the peak hip flexion moment and altered timing of the transition from flexion–extension moment) were reached by Lenaerts and colleagues [30]. Following a similar computational approach, Bartels and colleagues found a systematic HJC location error of 30 mm in the inferior direction and consequently, significant and substantial underestimation of the peak hip extension and abduction moment with respect to the image-based models (up to 23.1 and 15.8%) [29]. Significant differences in knee extension moment were also observed, but these were limited (4.9%). For hip rotation and ankle plantar flexion, differences in joint moments were negligible. The contradictory results between [101] and [102] can be explained by the different errors direction affecting the HJC estimates. In fact, whereas Lenaerts and colleagues [30] found that the estimated HJC was located about 30 mm anteriorly and 21 mm proximally, in the work of Bartels and co-workers [29], the errors in the HJC location were found mainly inferiorly (median value 18.7 mm) and posteriorly (median value 5.6 mm) and evenly spread along the medio-lateral axis.

Kirkwood and colleagues [103] considered four regressive methods, and found average maximum errors between 0.02 and −0.21 Nm/kg in the sagittal plane (0.12 to −1.26% BW * H), −0.05 to 0.27 Nm/kg in the frontal plane (−0.30 to 1.62% BW * H) and −0.05 to −0.07 Nm/kg in the transverse plane (−0.30 to −0.42% BW * H). Unfortunately, since the HJC location errors associated to the four regressive methods were not explicitly reported, a direct comparison among regressive methods is difficult. Similarly, Kiernan and colleagues [104] found, among the regressive methods analysed, maximum differences of approximately 0.1 Nm/kg (0.7% BW * H) in the hip extensor moment and hip abduction moment and no differences in the hip rotation moments. However, clinical statistically significant differences were found when computing the Gait Deviation Index Kinetic (maximum differences equal to 4.36 points with a threshold of clinical significance equal to 3.6 points) [101]. Sinclair and colleagues [102] found, during a squat exercise, statistically significant differences in both peak hip adduction moment and peak of knee external moment by comparing the HJC estimate as provided by a functional approach with those obtained from regression methods. Unfortunately, HJC locations were not reported.

#### Knee and ankle joints

Holden and Stanhope [105] found that an anterior knee joint centre (KJC) errors of ±10 mm caused, at fast gait speed, a maximum variation of the knee flexion–extension moment up to 0.71% BW * H. Furthermore, since knee joint moment decreases by decreasing the gait speed, at low speed KJC error can change the sign of the moment and thus impede the interpretation as flexor or extensor. Similar results were found by Reinbolt and colleagues [100] (errors on the knee flexion–extension peak equal to 0.92% %BW * H). Similar percentage errors were found for the abduction–adduction knee moment. Ankle joint moment errors were slightly larger for abduction–adduction than for dorsiflexion–plantarflexion. Bartels and colleagues [29] found small knee extension moment percentage differences between models (<5%) for a median deviation of the knee axis of 2.3°. For the ankle plantar flexion, differences in joint moments were negligible. Silva and Ambrosio [46] found in simulation a sensitivity of the knee moment similar to that observed for the application point of the external forces.

#### Multi-joint

Langenderfer and colleagues [47] found that joint moments were also most sensitive to an uncertain localisation of ALs near the joint, because it translates into variability in the joint centres identification (i.e. ankle moments were sensitive to location of the lateral malleolus, knee moments to the femoral epicondyle location, etc.). Reinbolt and colleagues [100] showed that the variability in the magnitude of the moments increased when moving from the ankle to the knee and hip joints. Largest RMS errors were observed for the hip flexion–extension and abduction–adduction moments (4.14 and 1.06% BW * H), followed by the knee flexion–extension moment mean (RMS error about 0.92% BW * H). Interestingly, when high-resolution bio-imaging techniques were employed for the personalization of the kinematic model, uncertainty in the joint parameters identification were greatly reduced [106, 107].

Besier and co-workers [94] found, over the ten subjects analysed, highly repeatable joint moment patterns for hip, knee, and ankle in both sagittal and frontal plane (R^2^ > 0.75). When joint parameters are functionally determined, slight improvements in the hip and ankle joint moments were observed. The statistical significant differences observed in the magnitude of the moments were explained by differences in the joint centre location and joint axes used in 2D and 3D.

### Inertial parameters

The motor tasks evaluated were: level walking (17 studies), walking on treadmill (2 study), and stair ascending/descending (2 studies). All studies except one [108] assessed the entire stride of the cycle (stance and swing phases).

Two types of investigation were performed (Table 3): in the 13 experimental studies, different set of BSIPs were used while in the 11 simulation studies the values of the BSIPs were varied applying deterministic or probabilistic approaches (for details see Table 3). Joint moment estimated with different values of BSIPs were compared and in same case the percentage variation was reported.

**Table 3:** Details of studies analyzed for the body segment inertial parameters (BSIPs) effect on the dynamics estimates

Study	Experimental	Simulation	Differences	Affect the dynamics (joint moments and powers)
[108]	1 RE [129] and 1 MI (MR)	NA	BSIPs were significantly different (exception: shank centre of mass, transverse—axis of radius of gyration about the knee)	Statistically significant differences in sagittal-plane of hip and knee moments and powers. Maximum of 0.3 and 0.7% BW * H/s for moment and power at the hip
[130]	2 RE (linear multivariate and non-linear) [131]	Variations up to 8%.	NA	Influence generally small. Propagation error at the hip joint due to errors at the knee and ankle
[26]	1 OM and 3 RE [14, 17, 22]	NA	BSIPs were significantly different (for the mass of the thigh Cheng closer to OM, for centre of mass Cheng and Dempster closer to OM, the moment of inertia no general results among body segment)	Differences in the sagittal plane of hip, knee and ankle moments. Differences smaller in the stance phase with respect to the swing phase
[132]	5 RE [12, 16, 20, 22, 131]	NA	NA	Appreciable differences on the sagittal plane of the hip moment. Some differences on frontal and coronal plane of the hip and knee moments (maximum 21.8% BW * H during stair descending). Larger during the swing phase
[114]	5 RE [12, 16, 20, 22, 131]	NA	NA	Small differences between ex vivo and in vivo data, between data from different populations and among different modality of inertial parameters acquisition. The root mean square value was negligible at the ankle and increased as moving proximally among the joints (maximum 21.8% BW * H in the transverse plane at the hip moment)
[20]	1MI (DXA-derived), 1 RE cadaver based [14]	NA	BSIPs were significantly different (maximum differences were found for the foot: mass 36% lower, centre of mass 15% more proximally, moment of inertia 47% lower)	Small effect during the stance phase, and more of an effect during the swing phase on joint moment on sagittal plane. Root mean square error increased moving proximally (at the hip 0.065 ± 0.043 Nm/kg body weight, corresponding approximatively to 0.4 ± 0.3% BW * H)
[21]	1MI (DXA-derived), 1 RE cadaver based [14]	NA	BSIPs were significantly different (maximum differences were found for moments of inertia 13–70%)	Negligible effect on joint moments of three children (sagittal plane), higher at the hip during swing phase (ranging from 0.03 Nm/kg body weight in the 10 year-old to 0.06 Nm/kg body weight in the 7 year-old, corresponding approximatively to 0.2% BW * H in the 10 year-old and 0.5% BW * H in the 7 year-old)
[31]	3 RE [14, 21, 133]	NA	BSIPs were significantly different (mass, centre of mass, radius and moment of inertis) for each segment for both the cerebral palsy and control groups	Negligible effect on joint moment for both groups (sagittal plane). Mean absolute variability between BSIP sets was low at the all three levels of the hip, knee and ankle (0.07, 0.04 and 0.01 Nm/kg respectively for the Control group and 0.04, 0.02 and 0.01 Nm/kg respectively for the CP group, corresponding approximatively to 0.5, 0.3 and 0.1% BW * H respectively for the control group and 0.3, 0.2 and 0.1% BW * H respectively for the CP group) No clinically meaningful difference between GDI-kinetic scores for the different BSIP protocols (maximum difference between BSIP sets was 2.4 and 2.8 points for Control and CP groups, respectively)
[47]	NA	Probabilistic analysis (advanced mean value, coefficients of variation: 0.12 for mass, 0.20 for moment of inertia, and 0.08 for centre-of-mass location ratio)	NA	Negligible effect of BSIPs on lower limb joint moments (stance phase, 3D), lower than the effect of anatomical landmarks definition. Variability in the magnitude of moments increased when moving from the ankle to the knee and hip
[111]	1 MI (DEXA), 4 RE [14, 22, 131, 134]	NA	BSIPs were significantly different: mass of the foot (up to 48.1%), centre of mass of thigh (8.3%), shank (9.7%) and foot, moment of inertia of shank and foot	Negligible effect of BSIPs on lower limb joint moments (3D), higher in swing phase and for hip joint (absolute difference less than 0.09Nm/kg (0.5% BW * H)
[112]	1 GM [19], 1 RE [16]	NA	BSIPs were significantly different (mass and longitudinal moment of inertia of the thigh)	Negligible effect of BSIPs on lower limb joint moments (root mean square difference 0.7 and 4.3% for the knee and hip, respectively. Difference lower than that caused by a 0.5 Hz adjustment in the cut-off frequency of the filter used to process the data
[117]	NA	Monte Carlo simulation (3000 iterations)	NA	BSIPs had a relatively small impact on lower limb joint moments compared to the impact of marker error (marker placement and soft tissue artifact). The only exception was hip flex/ext during the swing period
[110]	24 RE studies classified as: cadaveric, living (Caucasian), male living (Caucasian), female living (Caucasian), living (non-Caucasian)	Monte Carlo simulation 2000 iterations (Latin Hypercube, Sampling method)	BSIPs were significantly different between living subjects and cadaver studies (thigh, calf and foot masses up to 15.44%, centre of mass of the foot, and moment of inertia of thigh 36.65%), between Caucasian females and males (calf and foot masses, centre of mass of thigh and foot up to 15.05%, and moment of inertia of thigh 30.86%), and between Caucasian and non-Caucasian subjects (mass and moment of inertia of thigh 21.97%)	Simulation results showed effect limited to the swing phase of the knee and hip. Experimental results showed little effect on joint moments, except for the swing phase. The magnitude of difference in the swing phase due to variability in BSIPs is not much greater than the inter-trial variability. Distal BSIPs have little effect on proximal joint moment
[34]	NA	Statistical analysis (sample of errors from a normal distribution, three different maximum errors: 5, 10 and 15%)	NA	The refinement of the BSIPs has little effect in gait analysis results (compared to kinematic data and ground reaction force). Higher effect during the swing phase
[109]	5 RE [14, 15, 134], 1 GM [134]	BSIPs varied in steps over nine levels by a defined percentage (−40 to 40%) of the baseline value [14]	Significant differences were found for the mass, centre of mass and moment of inertia for both the leg and thigh segments (more than 40%)	BSIPS variations significantly affect most of the dynamic estimates (particularly the swing phase). The magnitude of these effects was generally less than 1% of body weight
[113]	5 RE [12, 14, 17, 131, 134], 1 GM [135]	NA	BSIPs were significantly different (from at least 9.73% up to 60%)	BSIPs variation has no effect on knee and ankle joint moments. For the hip joint moments, the effect is significant at slow, preferred and fast cadence (deviations reaching 17.91% and 20.11%, during the swing phase)
[100]	NA	Two sets of Monte Carlo analyses with and without noise (variations of BSIP values, together or separately, within 25, 50, 75, and 100% of their allowable bounds obtained from OM)	NA	BSIPs variation had no significant effect on calculated lower-extremity inverse dynamics joint torques (worst average 0.25% BW * H)
[39]	NA	BSIPs varied ±30% of the baseline values [12]	NA	Top-down approach: segment mass has effect on the calculated GRF. No report on lower limb joint moment
[35]	NA	Two sets of accuracy (baseline value [12]: variation of 5% and [20, 130, 136]	NA	Regarding the BSIPs, the only main contributor of the uncertainty in the joint torques was the foot mass, the remaining secondary contributors were the other BSIPs
[116]	NA	Perturbation of BSIP from 60 to 140% in steps of 10%	NA	Limited effect of an individual parameter perturbation on the calculated moments (largest effect is found for shank centre of mass, with a ratio of absolute difference in torque and relative parameter perturbation maximally 7.81 Nm for hip flexion moment, corresponding approximatively to 0.6% BW * H). The additional influence of perturbing two parameters simultaneously is small
[115]	1RE [14, 137]	Perturbation of mass and moment of inertia considering the uncertainty reported by Dempster [14]. Perturbation was performed considering the three segments (foot, shank, thigh) simultaneously and considering each segment independently	NA	Segment mass uncertainty has limited effect on the net joint moment. However, in case of adopted recursive method there is error propagation in proximal direction

For the experimental studies the type of BPISs estimation was specified: regression analyses (RE), geometric models (GM), data provided by means of medical imaging technologies (MI), and data obtained solving the non- linear optimization problem (OM)

Statistically significant differences were observed in the estimated BSIPs not only using different approaches [23] but also using different data within the same approach [109]. In a comprehensive analysis of 24 regression equation studies, BSIPs were found to be significantly different between living subjects and cadaver studies, between Caucasian females and males, and between Caucasian and non-Caucasian subjects, with highest differences for the moment of inertia of the thigh [110]. Comparing BSIPs values obtained with regression equations and geometric models, the estimated mass and the moment of inertia of the leg and the thigh can vary up to more than 40% [109]. Comparing BSIPs values calculated with dual energy X-ray absorptiometry and regression equation, the maximum difference was found for the foot segment and specifically for the moment of inertia [20, 111].

Analysing how these differences affect the lower limb joint moment estimations during activities of daily life, all studies reported smaller differences in stance compared to the swing phase, both during walking and stair ascending/descending (Table 3). Furthermore, all studies reported smaller effect moving distally from the hip to the ankle joint (Table 3). During walking, the maximum root mean square difference reported at the hip joint moment in the sagittal plane was for self-selected speed and fast-cadence 4.3% [112] and 20.11% [113], respectively. During stair ascending/descending, a maximum of 21.8% in the transverse plane at the hip moment was reported [114].

When the effect of the variation of a specific BSIP was investigated, no common result was found: in two cases the foot mass was the only main contributor of the uncertainty in the joint torques [35, 115], in another study the shank centre of mass has the largest effect [116] and in a third study distal BSIPs showed little effect on proximal joint moment [110].

Comparing the results with respect to other sources of error, the effect of BSIPs to the joint moment estimation was lower than that of anatomical landmarks definition [47], not much greater than that of the inter-trial variability [110], smaller than that of marker placement and soft tissue artifact [117], lower than that caused by a 0.5 Hz adjustment in the cut-off frequency of the filter used to process the data [112], and produced no clinically meaningful difference in the GDI-kinetic scores [31].

### Reliability, reproducibility and sensitivity analyses

A detailed report about reliability, reproducibility and sensitivity of joint moments is reported as additional material. While the single studies have been analysed in the previous sections, it is impossible at present to formulate a global or overall summary, because of a noticeable lack of standardisation in data analysis among Authors. This is unfortunate and calls for initiatives promoting recommended statistical indexes in the field.

## Discussion and conclusions

Uncertainty in the measured/estimated kinematics, anatomical calibration, and selection of appropriate joint model parameters, were confirmed as the main causes of errors in IDA results, with a potential serious impact in the clinical context. In addition, according to the limited available literature, the uncertainties in GRF measurement can have a comparable influence on the estimation of joint dynamics during gait. Looking at the role of BSIPs, results showed that, even if the effect of different BSIPs on joint moments was significantly different, it was not clinically meaningful for motor tasks of daily living such as walking, stair ascending/descending in healthy subjects.

The influence of the different mathematical approaches that can be adopted for the implementation of IDA was not analysed in the present review and this is a possible limitation. However, this aspect was deliberately excluded considering that differences in the computational implementations are meant to represent the same underlying mechanical system and are likely to have minor impact on clinical implementation of IDA results. Differences in estimated joint moments during gait [118] can produce maximal errors up to 25% of the range in flexion extension moment at the hip (negligible on other components), and this error can be regarded as minor for clinical use, when compared to other sources error that can result one order of magnitude higher, and when analysed considering how gait analysis data are interpreted in the clinics, which is comparing curves with coherent (in terms of calculation) reference bands, including inter- and intra-subject dispersion of data. In addition, the majority of IDA implementation for the calculation of joint moments for clinical use is made using the same commercial software, thus nullifying implementation differences.

The systematic analysis of the literature highlighted 67 papers discussing the sources of error affecting joint moments. The quality of the revised papers was evaluated, but no table was reported in the present work because quality is similar among studies: in all papers, the hypotheses were properly outlined and the overall design of the study was appropriate. However, a limited number of subjects was generally included, with effects on the grounding of the final conclusions. Most of these studies were indeed preliminary and/or explorative. The general criterion, followed by the authors of the present review, was only to exclude the works whose quality was considered insufficient and which did not add novel evidence.

The analysis of repeatability, reproducibility and sensitivity of moments across studies was also initiated and a comprehensive list of papers addressing each topic can be found in Additional file 1: Appendix S1 (on-line materials). However, the variety of parameters adopted in the literature is outstanding and preclude the formulation of a consistent overview, other than the analyses reported in the previous sections for kinematics, GRF, inertial parameters and joint model parameters.

Finally, further research is clearly needed to fully evaluate the uncertainties in GRF measurement, eventually analysing the problem in 3D, considering realistic in situ GRF errors, and evaluating the effect on external joint moments.

The literature highlights the importance of taking due care of:

1. Compensating for noise affecting marker trajectories especially when dynamics is estimated using kinematics only;
2. Performing the anatomical calibration, especially for the HJC, since the related uncertainty affects joint moments to a larger extent than other concurring factors. Uncertainties on HJC location between 10 and 30 mm have a great impact on both hip flexion–extension and hip abduction–adduction moments (maximum moment variation between −1.43 and 1.62% BW * H).
3. Identifying in a consistent manner joint parameter when comparing joint moments;
4. Interpreting IDA results in the light of the protocol used to estimate them and of the coordinate system used for their expression;
5. Properly assessing in situ GRF measurement errors, and estimating their potential effect on the final clinical decision process;
6. not extending the results found for walking and stair ascending/descending motor tasks in case of activities involving higher accelerations and when no ground reaction force is available (e.g. sprinting, kicking…) due to the larger effect on swing phase and proximal joints.

Therefore, the reader shall consider the potential benefits of using:

1. Functional joint centres, when hip calibration movements are not too difficult to perform for the subject and sufficiently large hip joint ranges of motion can be acquired (>30°) [119]. Care should be taken in limiting soft tissue artefacts during the functional tasks required for their estimate. The use of predictive equations is suggested otherwise [120];
2. Modified protocols that include additional markers or, for the foot, additional body segments that were proved to improve repeatability and\or the accuracy of joint moments and powers;
3. High-resolution bioimaging techniques can be successfully employed to produce personalized musculoskeletal models thus improving joint moment estimation and loading, provided that an adequate model is created;
4. Customized BSIPs to better highlight the muscle role in decelerating lower limb during the swing phase only in special populations, such as amputee patients [121–123].

It may be hoped that the large body of knowledge revised in this review can constitute further momentum to the standardization of the procedures to obtain and report joint moments, as already done by the International Society of Biomechanics in 2002 for the reporting of the joint kinematics [124, 125].

## Appendix

The Boolean strategy search applied for the three databases (Web of Science, Pubmed, Scopus) is reported.

**Table:**

General search
Web of Science: (Humans) AND (kinetic* OR inverse dynamics OR torque OR moment) AND (gait OR walking OR stair OR chair OR squat)
Pubmed: “Humans”[mesh terms] AND (“kinetics”[TIAB] OR “kinetic”[TIAB] OR “inverse dynamics”[TIAB] OR “torque”[TIAB] OR “torques”[TIAB] OR “moment”[TIAB] OR “moments”[TIAB]) AND (“gait”[TIAB] OR “walking”[TIAB] OR “stair”[TIAB] OR “stairs”[TIAB] OR “chair”[TIAB] OR “squat”[TIAB])
Scopus: TITLE-ABS-KEY (humans AND (kinetic* OR “inverse dynamics” OR torque OR moment) AND (gait OR walking OR stair OR chair OR squat))
REFINEMENT #1: KINEMATICS MEASUREMENTS AND PROCESSING
Web of Science: NOT (patholog* OR robot* OR exoskeleton OR wearable OR (upper limb) OR (upper body) OR (musculoskeletal model)) AND ((marker* movement) OR (marker* displacement) OR (marker* position) OR (skin motion) OR (skin movement) OR (soft tissue artifact) OR (soft tissue displacement) OR (anatomical calibration) OR (landmark) OR (anatomical point*) OR (marker placement) OR (joint angles) OR (kinematic errors) OR (kinematic consistency) OR (filter*) OR (protocol) OR (coordinate system*) OR (reference frame*) OR (anatomical frame*))
Pubmed: NOT ((“patholog*”[TIAB]) OR (“robot*”[TIAB]) OR (“exoskeleton” [TIAB]) OR (“wearable” [TIAB]) OR (“upper limb” [TIAB]) OR (“upper body” [TIAB]) OR (“musculoskeletal model” [TIAB])) AND ((“marker* movement” [TIAB]) OR (“marker* displacement” [TIAB]) OR (“marker* position” [TIAB]) OR (“skin motion” [TIAB]) OR (“skin movement” [TIAB]) OR (“soft tissue artefact” [TIAB]) OR (“soft tissue displacement” [TIAB]) OR (“anatomical calibration” [TIAB]) OR (“landmark” [TIAB]) OR (“anatomical point*”[TIAB]) OR (“marker placement” [TIAB]) OR (“joint angles” [TIAB]) OR (“kinematic errors” [TIAB]) OR (“kinematic consistency” [TIAB]) OR (“filter*”[TIAB]) OR (“protocol” [TIAB]) OR (“coordinate system*”[TIAB]) OR (“reference frame*”[TIAB]) OR (“anatomical frame*”[TIAB]))
Scopus: AND NOT (patholog* OR robot* OR exoskeleton OR wearable OR “upper limb” OR “upper body” OR “musculoskeletal model”)) AND (“marker* movement” OR “marker* displacement” OR “marker* position” OR “skin motion” OR “skin movement” OR “soft tissue artifact” OR “soft tissue displacement” OR “anatomical calibration” OR “landmark” OR “anatomical point*” OR “marker placement” OR “joint angles” OR “kinematic errors” OR “kinematic consistency” OR “filter*” OR “protocol” OR “coordinate system*” OR “reference frame*” OR “anatomical frame*”)
REFINEMENT #2: GROUND REACTION FORCES MEASUREMENTS AND PROCESSING
Web of Science: AND (ground reaction* OR force platform* OR centre of pressure OR dynamometric platform* OR contact force*) AND (error*)
Pubmed: AND (“ground reaction*”[TIAB] OR “force platform*”[TIAB] OR “centre of pressure” [TIAB] OR “dynamometric platform*”[TIAB] OR “contact force*”[TIAB]) AND ((“error*”[TIAB]) OR (“consistency”[TIAB]))
Scopus: AND (“ground reaction*” OR “force platform*” OR “centre of pressure” OR “dynamometric platform*” OR “contact force*”) AND error*
REFINEMENT #3: JOINT MODELS
Web of Science: AND (Joint Centre OR Hip Joint Centre OR Knee Joint Centre OR Ankle Joint Centre OR Joint Axis OR Knee Joint Axis OR Ankle Joint Axis OR dynamics model)
Pubmed: AND (“Center”[TIAB] OR “Centers”[TIAB] OR “Centre”[TIAB] OR “Centres”[TIAB] OR “axis”[TIAB] OR “axes”[TIAB] OR “dynamic model”[TIAB] OR “dynamic models”[TIAB])
Scopus: AND (“Joint Centre” OR “Joint Centres” OR “Hip Joint Centre” OR “Hip Joint Centres” OR “Knee Joint Centre” OR “Knee Joint Centres” OR “Ankle Joint Centre” OR “Ankle Joint Centres” OR “Joint Axis” OR “Joint Axes” OR “Knee Joint Axis” OR “Knee Joint Axes” OR “Ankle Joint Axis” OR “Ankle Joint Axes” OR “dynamics model” OR “dynamic model” OR “dynamic models”)
REFINEMENT #4: BODY SEGMENT INERTIAL PARAMETERS
Web of Science: AND (inertial parameter OR body segment parameter OR inertia OR moment of inertia OR center of mass)
Pubmed: AND (“inertial parameter” [TIAB] OR “body segment parameter”[TIAB] OR “inertia”[TIAB] OR “moment of inertia”[TIAB] OR “center of mass”[TIAB]))
Scopus: AND (“inertial parameter” OR “body segment parameter” OR inertia OR “moment of inertia” OR “center of mass”)

## Additional file

**Additional file 1:** **Appendix S1** reports the details and summary of the studies selected for analysis. Moreover, a specific description of the studies discussing repeatability, reproducibility and sensitivity of moments is also provided.
